# Two years follow-up study of the pain-relieving effect of gold bead implantation in dogs with hip-joint arthritis

**DOI:** 10.1186/1751-0147-49-9

**Published:** 2007-03-23

**Authors:** Gry T Jæger, Stig Larsen, Nils Søli, Lars Moe

**Affiliations:** 1 Department of Companion Animal Clinical Sciences, Norwegian School of Veterinary Science, Post Box 8146 Dep., N-0033 Oslo, Norway; 2 Department of Production Animal Clinical Sciences, Norwegian School of Veterinary Science, Post Box 8146 Dep., N-0033 Oslo, Norway; 3 Department of Food Safety and Infection Biology, Norwegian School of Veterinary Science, Post Box 8146 Dep., N-0033 Oslo, Norway

## Abstract

Seventy-eight dogs with pain from hip dysplasia participated in a six-month placebo-controlled, double-blinded clinical trial of gold bead implantation. In the present, non-blinded study, 73 of these dogs were followed for an additional 18 months to evaluate the long-term pain-relieving effect of gold bead implantation. The recently-published results of the six month period revealed that 30 of the 36 dogs (83%) in the gold implantation group showed significant improvement (p = 0.02), included improved mobility and reduction in the signs of pain, compared to the placebo group (60% improvement).

In the long-term two-year follow-up study, 66 of the 73 dogs had gold implantation and seven dogs continued as a control group. The 32 dogs in the original placebo group had gold beads implanted and were followed for a further 18 months. A certified veterinary acupuncturist used the same procedure to insert the gold beads as in the blinded study, and the owners completed the same type of detailed questionnaires. As in the blinded study, one investigator was responsible for all the assessments of each dog. The present study revealed that the pain-relieving effect of gold bead implantation observed in the blinded study continued throughout the two-year follow-up period.

## Background

Hip-joint arthritis, mostly from canine hip dysplasia (CHD), is a common, non-curable and painful disease amongst medium and large breed dogs [1-4] and therapy is palliative at best [5].

Implantation of gold beads in both humans and animals with arthritis was first attempted by veterinary traditional acupuncturists [6]. Other researchers [7,8] who implanted gold beads in dogs, observed no clinical effect after six- and three-months study periods. However, in a recent paper we revealed that gold bead implantation had a significant pain-relieving effect in a six months controlled, double-blinded clinical trial [9]. The initial mean pain scores in the gold-bead implantation and control groups were 5.6 and 4.8, respectively, where 0 was no pain and 10 was extreme pain. After 3 months the mean pain scores were significantly reduced in both groups. After six months, no further reduction had taken place in the control group, while a significant further reduction in mean pain score (to 1.9) was found in the gold group. The total reduction in mean pain score was in the gold group 65% compared to 36% in the placebo group (p < 0.01). Each dog's overall response, according to its owner's impression of change in mobility, lameness, stiffness and behaviour at home, scored on a six-point Likert scale, showed a significant improvement (p = 0.02) in 30 of the 36 dogs (83%) in the gold implantation group compared to 60% improvement in the placebo group [9].

The aim of the present study was to evaluate whether the significant improvement that we found after six months was still present 24 months after gold bead implantation.

## Materials and methods

### Study design

The study was carried out as a randomised, placebo-controlled and double blind clinical trial with stratified parallell group design for six months (Jaeger and others 2005), and then as an open follow-up study for 18 months. All dogs with a history of pain and/or lameness or dysfunction of the hind limbs due to hip dysplasia and with no previous acupuncture history were invited to the study. The diagnosis of hip dysplasia was based on radiographs and was graded as mild, moderate or severe according to the guidelines of the Scientific Commission of the Nordic Kennel Union, and Federation Cynologique Internationale. The dogs were owned privately and lived in private households with their owners for the entire trial period, and none of the owners had more than one dog in the trial.

The treatment was blinded for both the owners and the responsible clinical investigator during the first six months of the trial period. The randomisation code was then broken and the placebo-treated dogs were offered gold bead implantation, after which the open study followed. The implantation procedure was identical to that used at the commencement of the trial. The dogs were divided into three groups (Table 1). The GG group consisted of dogs treated with gold bead implantation from Day 0 and followed for a total of two years. The PG group was formed from dogs that were initially in the placebo group, were treated with gold bead at six months and then followed for a further 18 months. The PC group was initially in the placebo group and was followed for a further 18 months without any gold bead implantation.

**Table 1:** Number of dogs (n) with mild/moderate or severe hip dysplasia divided into three different weight groups, where the hip status and body weights are used as stratification factors.

Treatment group	Number of dogs (n)	Drop Out (n)	Weight group (kg)	Mild or Moderate hip dysplasia (n)	Severe hip dysplasia (n)	Total (n)
GG	36	2	≤ 20.0	1	3	4
			20.1 – 34.9	8	8	16
			≥ 35.0	8	6	14
PG	33	1	≤ 20.0	0	1	1
			20.1 – 34.9	7	9	16
			≥ 35.0	10	5	15
PC	9	2	≤ 20.0	0	0	0
			20.1 – 34.9	4	0	4
			≥ 35.0	0	3	3

Total	78	5		38	35	73

The GG group = gold implantation from Day 0 to 24 months, the PG group = placebo treatment the first six blinded month and then 18 months gold bead implantation, PC group = placebo treatment the first six blinded month and as a control group the next 18 months

### Animals

In the double-blind study 38 and 42 dogs were allocated to gold bead implantation and to placebo treatment, respectively [9,10]. During this period two dogs from the gold implantation group discontinued for reasons unrelated to the treatment (drop outs). After the randomization code was broken, 73 of the 78 dogs that completed the six month study were followed for further 18 months; the present study. Of the 42 dogs in the placebo group in the six-month study, 33 received gold bead implantation and nine continued in the 18-month follow-up period as a control PC group (Table 1). The 18-month follow-up groups consisted of 30 males and 43 females, with a mean age and weight of 6.3 years and 35.5 kg. The mean duration of gold bead implantation was 21.6 months at the end of the study. Two of the 36 dogs originally treated with gold implantation (GG group) dropped out and two were euthanized on the owners' request due to insufficient pain-relieving effect (withdrawals) during the study period. Of the 33 dogs in the PG group, one dropped out, two were euthanized (withdrawals) and one was withdrawn for unknown reasons. Of the nine dogs in the PC group, two dropped out.

### Clinical procedure

An IVAS (International Veterinary Acupuncture Society)-certified acupuncturist performed the gold bead implantation. The same investigator as in the six-month blind trial was responsible for the follow-up period and performed all assessments of each dog. Owners were questioned about their dog's clinical signs and medical history using the same standardized questionnaire as for the six-month blinded period. The same examination procedure including videotaping the dogs in five different gaits was used. Hip radiographs and blood samples were taken at the end of the 18-month follow-up period.

The veterinarian's assessment of pain was based on the response to rotation, flexion and extension of the affected hip, and graded on a 4-point scale, where "no pain response" = 1, "mild pain response", tries to move away = 2, "moderate pain response", turns head toward the hip, slight vocalization = 3 and "large pain response", turns head with intention to bite, howls = 4 [11]. Each hip was given a separate score and then both added to a total hip score.

During assessment, each dog was videotaped walking, trotting before and after stretch/extension of each hip, and performing left and right turns. Lameness was graded on a 5-point scale for each gait and scored as "no lameness" = 0, "barely disturbed locomotion" = 1, " locomotion disturbed but limb(s) still bearing weight" = 2, "lameness with limb(s) not always bearing weight" = 3, "no weight bearing on limb(s)" = 4 [12]. A total lameness score was calculated by adding the scores for the four gaits at each examination.

Each dog's overall response, derived from to its owner's general impression of change in mobility, lameness, stiffness and behaviour at home and during different types of exercise, was scored on a six-point Likert scale [13], where "large deterioration" = 1, "mild deterioration" = 2, "no change in signs" = 3, "mild improvement" = 4, "large improvement" = 5 and "without any signs of hip dysplasia" = 6.

The owners were asked to assess their dog's quality of life, taking into consideration its ability to fulfil its physical, mental and social needs, and signs of pain and dysfunction, as poor, fairly good or very good.

The owners were asked to give their opinions of the pain-relieving effect of gold bead implantation, regardless of the duration of effect, as one of four alternatives: no effect, small effect, good effect or very good effect.

### Use of palliative medication

Owners were allowed to administer other forms of palliative treatment if their dog needed it. In such cases only non-steroid anti-inflammatory drugs (NSAIDs) were used and it was recorded.

### Statistical analysis

Dogs that discontinued the study due to reasons related to the treatment (withdrawals) were included in the analysis, using the procedure of last observation carried forward.

All assumed continuously-distributed factors and variables were expressed as mean values with 95% confidence interval (CI) calculated in accordance with the Student procedure [14]. Discontinuously-distributed factors and variables were expressed in contingency tables [15].

Comparisons of groups with regard to assumed continuously-distributed variables were performed with Analysis of Variance (ANOVA), with repeated measurements and initial scores as covariate [16]. Matched-pairs ANOVA model was used for analysis within groups.

For change within groups, cross-table analysis was performed [15].

All comparisons between groups, and changes within groups, were performed two-tailed with a significance level of 5%.

## Results

The mean hip-pain score recorded by the clinical investigator in the pooled gold implantation group was significantly reduced (p < 0.01) from day 0 to 24 months (Table 2). In the PC group an increased mean hip-pain score was detected in the same period, although this was not significant (p = 0.28). The change in mean hip pain score was significantly larger in the pooled gold implantation group compared to the PC group (p = 0.012).

**Table 2:** Mean hip-pain score and mean lameness score (95% CI) at Day 0 and 24 months in the pooled gold implantation group evaluated by the clinical investigator.

	Time	GG +PG (pooled) (n = 66)	PC (n = 7)
	
Pain score	Day 0	4.8 (4.5 – 5.2)	4.4 (2.9 – 5.9)
	24 months	4.2 (3.8 – 4.6)	5.0 (3.9 – 6.1)
Lameness score	Day 0	1.9 (1.3 – 2.6)	2.6 (0.6 – 4.6)
	24 months	1.2 (0.5 – 1.8)	3.0 (0.7 – 5.3)

For abbreviations see Table 1

The mean lameness score observed by the clinical investigator was reduced from day 0 to 24 months in the pooled gold bead implantation group (p = 0.09) and numerically increased in the PC group (p = 0.84) in the same period (Table 2). The change in mean lameness score between the groups was not significant. Thirty-four of the 66 dogs in the pooled gold bead implantation group were initially recorded as lame, with a total lameness score of 127. After 24 months, the number had decreased to eighteen dogs with a total lameness score of 76. In the PC group, five of seven dogs were initially recorded as lame, with a total lameness score of 18; after 24 months two dogs were still lame, but had increased the total lameness score to 21.

The changes reported by the owners more or less mimicked those recorded by the clinical assessor. The hip-pain score in the pooled gold implantation group, reported by the owners, was significantly reduced (p < 0.01) during the 24 months period (Table 3). A reduction was also detected in the PC group, but this was not found significant (p = 0.06). The reduction in the pooled gold implantation group was larger then in the PC group, but the difference was not significant (p = 0.13).

**Table 3:** Owners' assessments of hip pain and dysfunction using a 10 cm visual analog scale from 0 – 10 where 0 = no pain or dysfunction and 10 = extreme pain or dysfunction. The results are expressed as mean values with a 95% confidence interval.

	Time	GG +PG (pooled) (n = 66)	PC Group (n = 7)
	
Pain signs	Day 0	5.1 (4.8 – 5.4)	4.4 (3.4 – 5.5)
	24 months	2.4 (1.8 – 2.9)	3.0 (1.2 – 4.8)
Dysfunction	Day 0	4.3 (3.9 – 4.7)	3.9 (3.0 – 4.7)
	24 months	2.2 (1.6 – 2.7)	4.0 (2.3 – 5.7)

For abbreviations see Table 1

The degree of dysfunction, reported by the owners, was significantly reduced (p < 0.01) in the pooled gold implantation group during the study (Table 3). No change was detected in the PC group (p = 0.74), and the reduction in dysfunction in the pooled gold implantation group was found to be significantly larger than in PC group (p < 0.01).

Table 4 shows the changes (improvement or deterioration) in the dog's behaviour from hip-pain, according to the owner's impression, for the different treatment groups. A significant improvement in the signs of hip-pain (p < 0.01) was found in the pooled gold bead implantation group, compared to the PC group in the period from day 0 to 24 months. The prevalence of dogs in the pooled GG and PG groups that demonstrated improvements after 24 months of treatment was 81.8% (CI 70.4 – 90.2) (Table 4). The improvement was found to be less in the PG-group compared to the GG-group (p = 0.05). However, the PG-group showed a significant better improvement compared to the PC group (p = 0.03).

**Table 4:** Number of dogs treated with gold implantation and a control group that showed changes in the signs of hip dysplasia, according to their owners' general impressions of their dog's behaviour in its daily life after 24 months of treatment.

Treatment group	No pain signs	Large improvement in signs	Moderate improvement in signs	No change in signs	Moderate deterioration in signs	Severe deterioration in signs	Total
GG	3	19	10	0	2	0	34
PG	1	12	9	2	5	3	32
GG+PG (pooled)	4	31	19	2	7	3	66
PC (control)	0	0	1	2	4	0	7

For abbreviations see Table 1

The scores for overall hip-pain improvement or deterioration according to the owner's impression of their dog's behaviour after six months and 24 month for the GG group is given in Table 5. Owner-reports indicated that 85.3% (CI 68.9 – 95.1) of the dogs with gold bead implantation from Day 0 showed improvements in the overall hip-pain score after six months of treatment. After 24 months, 94.1% (CI 80.3 – 99.3) of the dogs with gold bead implantation showed improvements, but the increase was not found statistically significant. It can be seen from the Table 5 that 15 dogs (the bold numbers) were unchanged in their hip-pain scores when the scores at six and 24 months were compared. Nine dogs showed a further improvement from six to 24 months, eight dogs had reduced improvements and two dogs showed deterioration.

**Table 5:** Number of dogs in a cross table with different scores for the overall hip pain improvement or deterioration according to the owners' general impression of their dog's behaviour in its daily life after six and 24 months in the originally gold implantation group.

		Evaluation of hip pain signs after 24 months						
		
	Owner's assessment → ↓	No pain signs	Large improvement in signs	Moderate improvement in signs	No change in signs	Moderate deterioration in signs	Severe deterioration in signs	Total
		
Evaluation of hip pain signs after 6 months	No pain signs	**1**	3	1	0	0	0	5
	Large improvement	2	**10**	4	0	0	0	16
	Moderate Improvement	0	4	**4**	0	0	0	8
	No change	0	2	1	**0**	2	0	5
	Moderate deterioration	0	0	0	0	**0**	0	0
	Severe deterioration	0	0	0	0	0	**0**	0
	Total	3	19	10	0	2	0	34

The last row of the table shows the situation after six months, and the last column the results after 24 months. The bold figures display no change in hip pain signs or dysfunction from six months to 24 months. Figures to the right and left of the bold figures express the deterioration and ameliorations, respectively.

In Table 6 the owners' assessments of their dogs' quality of life after 24 months is shown. The prevalence of "very good" quality of life in the pooled gold bead implantation groups was 63.6% (50.9 – 75.1), while the corresponding value for the GG group was 70.6% (52.5–84.9) and for the PC group 71.4% (29.0–96.3).

**Table 6:** Owners assessments of their dog's quality of life after 24 months in the pooled (GG+PG) group treated with gold bead implantation and in the control group.

Quality of life after 24 months				
Treatment group	Very Good	Fairly good	Poor	Total

GG/PG (pooled)	42	18	6	66
GG	24	7	3	34
PC (control)	5	2	0	7

For abbreviations see Table 1.

When asked their opinion of the pain-relieving effect of gold bead implantation, 100% (CI 89.7 – 100.0) of owners of dogs in the GG group reported a good or very good effect, while the corresponding value for the PG group was 78.1% (CI 60.0 – 90.7). Small or no effect was reported by 21.9% (CI 9.3 – 40.0). Pooling the two gold implantation groups produced a value of 89.4% (CI 79.4 – 95.6) of owners that reported good or very good effect.

At the termination of the study, or at time of death, the owners were asked if they had observed a positive pain-relieving effect of the gold bead implantation, and if so, for how long. Continuous pain-relieving effect of gold bead implantation was reported in 79.7% (CI 68.3 – 88.4) of the dogs. If a dog went with gold implantation for 20 months for example, and showed a positive effect for 18 of these months, the recorded duration of effect was 90%. The mean duration of effect for gold-implanted dogs was 90.7% (CI 87.5 – 94.1; range 17.5% to 100.0%).

Use of NSAIDS was recorded in the period between six and 24 months. Three of 34 dogs (CI 1.9 – 23.7) in the GG group reported use of NSAIDs periodically or daily after appearance of pain signs, whereof one dog was withdrawn. Seven of 32 dogs (CI 9.3 – 40.0) in the PG group reported use of NSAIDs periodically or daily after appearance of pain, whereof two dogs were withdrawn. Two of seven dogs in the PC group reported use of NSAIDs periodically or daily. No significant difference was detected between these groups.

## Discussion

The same pain-relieving effect that was revealed in the blind six-month study [9] continued in the open study of gold implantation, as assessed by both the owners and the clinical investigator. The result is very promising, but not unexpected on the basis of the results from the six-month blinded study. It is, however, remarkable that a one-time implantation of gold beads should have a sustained pain-relieving effect over so many months.

This clinical trial was originally an attempt to model the pain-relieving effect of gold-bead implantation in humans with degenerative arthritis. In humans, a two-year period of medical follow-up is rather brief, seen in relation to presumptive duration of life. Dogs with a mean lifespan ten times less than man [17], undergo similar age-related diseases. The expected lifespan of a newborn dog is 5–7 years [17] and two years represents roughly 25% of its expected duration of life.

The largest weakness with the present study is that it was non-blinded during the last 18 months, with both the owner and the clinical investigator being aware of the treatment. The results may therefore have been biased in a positive direction by the evaluators, as in any other non-blinded clinical trials [18], and the clinical evaluation of improvement or deterioration will to a certain degree be affected by the owner's enthusiastic or disappointed attitude. To reduce this possible bias, standardized and carefully-designed questionnaires were used.

The pain-relieving effect in the 24 months follow-up study that was recorded by both the owner and the clinical investigator was, however, slightly smaller than in the blind six months study. Perhaps the owners belonging to the originally placebo group were more sceptical since a majority of them had earlier believed that their dogs had received gold implantation, and now reported more cautious results [10]. This is in accordance with other studies [19,20], since there is a strong placebo effect in the blind study and probably a nocebo effect in the open study.

Non-blinded studies usually over-estimate treatment effect by about 17% [21]. However, the present study is not comparable with a simple non-blinded study, since this study started as a blinded study that later crossed over to a non-blinded study.

Another limitation with the present study is that the control group without gold implantation was small and consisted probably of dogs whose owners were satisfied with their animal's clinical performance and level of pain. To entice owners to complete the study, the owners of the 42 dogs in the original placebo group were promised gold bead implantation when the randomization code was broken, if their dogs still had pain signs due to hip-joint arthritis. Thirty-three owners accepted gold bead implantation, leaving only nine dogs in the subsequent control group, and two of these dropped out. It is possible that the remaining dogs in the control group had minimal pain signs and therefore represent a selection bias. If so, the differences in pain between the pooled gold-bead implantation group and the control group could be expected to be smaller than in a double blind study.

There were five withdrawals from the pooled gold implantation group in the follow-up study, due to inadequate pain-relieving effect. These dogs were included in the analysis of the results.

Pain-relieving medications were only used by approximately 16% of the dogs and equally distributed in all groups, and likely not to have influenced to the overall results.

The cause and the mechanisms of the pain-relieving effect were not investigated in the present study and need to be explored in detail in future studies.

## Conclusion

A positive, long-term and sustained palliative effect of gold-bead implantation in dogs with hip dysplasia has been demonstrated, which needs to be confirmed in a new, placebo-controlled blind study.

## Competing interests

The author(s) declare that they have no competing interests.

## Authors' contributions

GJ conceived the idea of the study, participated in the planning, examined all the dogs and interviewed the owners. She treated most of the dogs in the open study, collected and computed the data, and wrote the manuscript.

LM and NS took part in the conception of the study, its planning and the clinical design. They participated in writing the tables and the manuscript. LM was the leader of the project.

SL participated in the design of the study and performed the statistical analyses.

All authors approved the final manuscript.
